# Ambroxol Improves Neuronal Survival and Reduces White Matter Damage through Suppressing Endoplasmic Reticulum Stress in Microglia after Intracerebral Hemorrhage

**DOI:** 10.1155/2020/8131286

**Published:** 2020-03-26

**Authors:** Xuheng Jiang, Ji Zhang, Bojin Kou, Chao Zhang, Jun Zhong, Xuanyu Fang, Xiaofei Huang, Xiaojun Zhang, Fangke Xie, Quan Hu, Hongfei Ge, Anyong Yu

**Affiliations:** ^1^Department of Emergency, The First Affiliated Hospital of Zunyi Medical University, 563003 Zunyi, Guizhou, China; ^2^Department of Neurosurgery and Key Laboratory of Neurotrauma, Southwest Hospital, Third Military Medical University (Army Medical University), 400038 Chongqing, China

## Abstract

Intracerebral hemorrhage (ICH) has been becoming a serious public health problem. Pneumonia, occurring in 43% of all ICH patients, is a common complication heavily influencing outcome and accounting for more than 1/3 of the overall mortality in patients with ICH. Ambroxol may be an effective additional treatment for ICH patients with pneumonia. But its effect and potential mechanism on functional recovery post-ICH still remain elusive. In the present study, the results indicated that 35 mg/kg and 70 mg/kg ambroxol facilitated neuronal survival and reduced white matter fiber bundle damage due to mitigating microglial activation and reducing proinflammatory cytokine accumulation in mice with ICH. The possible mechanism might be due to suppressing endoplasmic reticulum stress involving the IRE1*α*/TRAF2 signaling pathway, which paves a new path for the treatment of ICH and opens a new window for the use of ambroxol in clinical practice.

## 1. Introduction

Intracerebral hemorrhage (ICH) has been becoming a serious public health problem, which accounts for 10–20% of all strokes worldwide and is associated with high rates of death and disability [1, 2]. Pneumonia, occurring in 43% of all ICH patients, is a common complication heavily influencing outcome and accounting for more than 1/3 of the overall mortality in patients with ICH [3, 4]. Except for antibiotic treatment, ambroxol has been proven to be an effective airway humidification liquid, which improves the patient's lung function through promoting the synthesis and secretion of pulmonary surfactant and diluting sputum to facilitate pulmonary ventilation dysfunction, thereafter, inhibiting further pulmonary damage after severe brain injury [5]. Hence, ambroxol was usually employed to protect lung against infection after ICH. What is more, previous research has demonstrated that oral administration of ambroxol helps increase brain glucocerebrosidase (GCase) activity to reduce alpha-synuclein protein levels in healthy nonhuman primates [6], which provides further evidence for clinical trials of ambroxol in the treatment of patients with Parkinson's disease [7, 8]. In addition, a recent investigation has shown that ambroxol facilitates and protects motor units, improves axonal plasticity, and extends overall survival via inhibiting nonlysosomal (GBA2) glucocerebrosidase activity in the spinal cord with amyotrophic lateral sclerosis (ALS) in the SOD1^G86R^ mice [9]. However, the effect of ambroxol on brain recovery and potential mechanism still remains elusive.

Ambroxol, used for pneumonia, has multifaceted pharmacological benefits such as mucokinetic and mucociliary effects, anti-inflammation, antioxidant, and surfactant stimulation [10]. Furthermore, study also demonstrates that ambroxol helps decrease levels of lipopolysaccharide induced by lung hemorrhage, edema, exudation, infiltration with neutrophils, and release of cytokines in animal with acute lung injury [11]. Meanwhile, previous reports have proven that ambroxol decreases the levels of proinflammatory factors such as TNF-*α*, IL-1*β*, IL-6, and IL-8 in rats with lung infection [10, 11]. In addition, previous study has indicated that ambroxol can effectively cross the blood-brain barrier (BBB) and has no harmful effect even at high doses [6, 7, 12]. Microglia, acting as guardians of the brain, are recognized to be the first nonneuronal cells to respond to various acute brain injuries [13], including ICH [14]. Meanwhile, evidence has showed that activated microglia are the primary source of chemokines, cytokines, proteases, prostaglandins, ferrous iron, and other immunomodulatory molecules in the brain [14]. Thereafter, whether ambroxol could regulate microglial activation and the potential mechanism is worth elucidating after ICH.

In the present study, we hypothesized that ambroxol benefits functional recovery via modulating microglial activation in mice with ICH. Our results indicated that 35 mg/kg and 70 mg/kg ambroxol facilitated neuronal survival and reduced white matter fiber bundle damage due to mitigating microglial activation and reducing proinflammatory cytokine accumulation in mice with ICH. The possible mechanism might be due to suppressing endoplasmic reticulum stress involving IRE1*α*/TRAF2 signaling pathway. The present study paves a new path for the treatment of ICH and opens a new window for the use of ambroxol in clinical practice.

## 2. Materials and Methods

### 2.1. Animals

Adult male C57BL/6 mice (22–26 g) were purchased from the laboratory of Third Military Medical University. All experiments were performed according to the China's animal welfare legislation for protection of animals used for scientific purpose and were approved by the local authorities of Third Military Medical University for the use of laboratory animals. All mice used in the present study were given ad libitum access to food and water.

### 2.2. Experimental Design and Intracerebral Hemorrhage (ICH) Model

**Figure 1:**
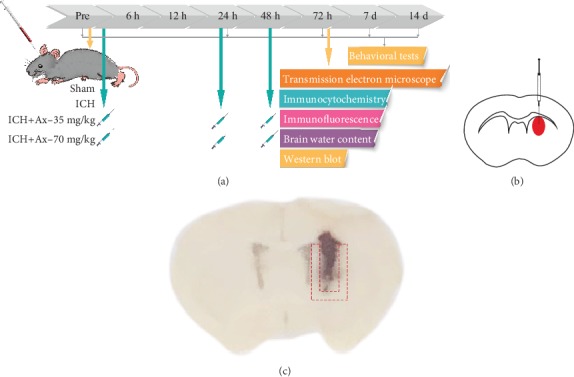
The study design and samples for experiments. (a) The time course of study design. (b) The schematic of the experimental ICH model. (c) The dotted outline around the hematoma was the region for next experiments.

The study design is shown in Figure 1(a). The ICH model was established in accordance with previously described methods as shown in Figure 1(b) [15]. Mice were anesthetized with isoflurane (3% for induction and 1-1.5% for maintenance). Then, they were immobilized on the stereotaxic frame. A small cranial burr hole was performed at the frontal skull under stereotactic guidance with a precise location (bregma coordinates: 0.8 mm anterior and 2 mm lateral to the midline). A total of 25 *μ*L autologous blood was injected 3 mm deep into the right basal ganglia at a rate of 2 *μ*L/min using a microinfusion pump. Thereafter, mice were randomly assigned and matched for body mass to the following 4 groups: sham, ICH + vehicle (ICH), ICH + Ax‐35 mg/kg, and ICH + Ax‐70 mg/kg. All experiments and analyses were performed by individuals blinded to treatment groups.

### 2.3. Drug Administration

Ambroxol hydrochloride was purchased from Sigma-Aldrich (St. Louis, MO) and dissolved in saline. For treatment groups, mice intraperitoneally (IP) received 35 mg/kg or 70 mg/kg ambroxol hydrochloride immediately and 24 h after ICH, followed by once a day for 2 days. For the ICH group, mice received an equal volume of saline at the same time point as treatment groups. In the sham group, mouse received saline only.

### 2.4. Brain Water Content Examination

The brain water content of each group was measured as an indicator of cerebral edema after 48 hours. Briefly, the hemorrhagic lateral basal ganglia were removed, and the tissue was weighed using an accurate electronic balance to determine the wet weight after anesthesia. The brain sample was dried at 100°C for 24 h and weighed again to obtain a dry weight. The brain water content was calculated using the formula ((wet weight − dry weight)/wet weight) × 100%.

### 2.5. Behavioral Tests

The rotarod test was performed as described by our previous work [16]. The speed was set to increase gradually from 5 to 35 rpm, and the latency to fall (or cling to and spin with the rod for three full rotations) within 3 min was recorded for statistical analysis. Three trials for each mouse were performed separated by 10 min. A latency less than 60 s per day before implementing ICH was set as an exclusion criterion for surgery.

The adhesive removal test was performed by an observer independently as previously described [2]. Before the actual test, the mice were trained once a day for 3 days before surgery to familiarize them with the test. The mice were placed into a transparent box for 3 min to get used to the new environment. Then, the adhesive tape (2 mm × 2 mm) was pasted on the affected side forepaw. The time to remove the adhesive tape was measured with a cutoff of 120 s.

### 2.6. Western Blotting

Western blotting was performed as previously described [17]. Samples (shown in Figure 1(c)) were harvested, and proteins were extracted by lysing 2 mm tissue (basal ganglia) around the hematoma on ice in RIPA (Sigma-Aldrich, St. Louis, MO, USA) supplemented with protease and phosphatase inhibitors (Roche, Indianapolis, IN, USA). Protein concentration was measured by an enhanced BCA protein assay kit (Beyotime, Beijing, China). Protein samples were then separated by 10% or 15% SDS-PAGE electrophoresis and transferred to a polyvinylidene difluoride membrane (PVDF). The PVDF membranes were blocked in TBST (0.5% Tween-20 in Tris-buffered saline) containing 5% (*w*/*v*) nonfat dry milk for 1 hour, and the primary antibody was incubated overnight at 4°C, including anti-CHOP antibody (1 : 1000, Cell Signaling Technology, Danvers, MA), anti-IL-1*β* antibody (1 : 1000, Cell Signaling Technology, Danvers, MA), anti-TNF-*α* antibody (1 : 1000, Cell Signaling Technology, Danvers, MA), anti-TRAF2 antibody (1 : 1000, Abeam, Cambridge, UK), anti-IRE1*α* (1 : 1000, Proteintech Group, Inc, Beijing, China), anti-*β*-actin (1 : 5000, Proteintech Group, Inc, Beijing, China), and anti-Tubulin (1 : 5000, Proteintech Group, Inc, Beijing, China). The PVDF membranes were treated with the relevant secondary antibody (1 : 5000) for 1 hour at room temperature. All membranes were detected by ChemiDoc^TM^ XRS^+^ imaging system (Bio-Rad, Berkeley, California, USA) using the WesternBright ECL kit (Advansta, Menlo Park, CA, USA). The optical density image of each film was measured using Image Lab^TM^, and the image was quantified by using ImageJ.

### 2.7. Immunohistochemistry and Hematoxylin and Eosin (HE) Staining

The mice were anesthetized and perfused with 0.1 M PBS (pH 7.4) and 4% paraformaldehyde. The brain was removed and kept in the 4% formaldehyde for 4 hours and then immersed in 30% sucrose at 4°C until sinking. The brain was cut into 4 *μ*m thick sections using a paraffin slicer (CM2235, Leica, Germany) and partially stained with HE. For immunohistochemistry, brain sections were dewaxed and antigen-repaired according to the standard procedures. Then, sections were incubated in endogenous peroxidase for 10 min. After blocking with normal goat serum or with 0.5% v/v Triton-X 100 (Sigma-Aldrich, St. Louis, MO) in PBS. Samples were incubated in primary antibodies anti-CD16/32 (1 : 200, BD Pharmingen, San Jose, CA, USA) and NeuN (1 : 400, Abeam, Cambridge, UK) for 16-18 h at 4°C. After washing, they were incubated in horseradish peroxidase- (HRP-) conjugated goat anti-mouse immunoglobulin G (ZSGB-BIO, Beijing, China). Then, the 3-diaminobenzidine (DAB) kit was employed to stain in color. Sections were counterstained with hematoxylin and dehydrated with ethanol and xylene to prep for mounting. Thereafter, coverslips were mounted onto glass slides. Then, partially dewaxed and antigen-repaired, after 10 min endogenous peroxidase blocked incubated, anti-CD16/32 (1 : 200, BD Pharmingen, San Jose, CA, USA), NeuN (1 : 400, Abeam, Cambridge, UK) were incubated at room temperature for 2 hours, and after incubation of second antibody was goat anti-mouse immunoglobulin G (ZSGB-BIO, Beijing, China), 3-diaminobenzidine (DAB) was developed in color. Images were captured using a scanning microscope (Olympus, Tokyo, Japan*).*

### 2.8. Immunofluorescence

After anesthesia with isoflurane, the mice were perfused with 4% paraformaldehyde. The mouse brain was obtained to make into 25 *μ*m freezing slices. After fixed with 0.25% Triton x-100 (Sigma-Aldrich) in PBS for 30 min, the sections were sealed by 5% bovine serum albumin (BSA) for 2 hours and then incubated overnight at 4°C. Antibodies include anti-CD16/32 (1 : 200, BD Pharmingen, San Jose, CA, USA), anti-CHOP (1 : 400, Cell Signaling Technology, Danvers, MA), and anti-MBP (1 : 400, Santa Cruz Biotechnology, CA, USA). The corresponding fluorescent secondary antibody was then incubated for 2 hours at room temperature, and the nuclei were counterstained with DAPI for 10 minutes. Images were captured by a confocal microscopy (LSM780; Carl Zeiss, Weimar, Germany) and examined by Zen 2011 software (Carl Zeiss, Weimar, Germany).

### 2.9. Transmission Electron Microscopy (TEM)

TEM was performed as previously reported [18]. The mice were anesthetized with isoflurane (3% for induction, 1-1.5% for maintenance), and brains were rapidly removed after washing with 0.9% saline. The brain samples (about 1 mm^3^) around hematoma were obtained and incubated in 4% paraformaldehyde. Thereafter, samples were incubated in 2% glutaraldehyde overnight at 4°C and then transferred to 1% citric acid for fixation. Then, they were dehydrated with gradient acetone after soaking in uranyl acetate. Thereafter, samples were embedded with epoxy resin and sliced into 70-90 nm. They were counterstained with lead citrate after placing on the copper trough grid, and the ultrastructure of nerve tissue was observed under transmission electron microscope. After anesthesia, the mice were perfused with 4% paraformaldehyde, and the edge tissue of the hematoma was obtained for about 1 mm^3^; 2% glutaraldehyde was fixed and then transferred to 1% citric acid for fixation, uranyl acetate soaked, acetone gradient dehydrated, epoxy resin embedded, and sliced into 70-90 nm, then placed on the copper trough grid, counterstained with lead citrate, and the ultrastructure of nerve tissue was observed under a transmission electron microscope. The G-ratio of myelinated fibers was calculated by using ImageJ software as a ratio of axonal diameter to axon diameter + myelin, and 100 myelinated fibers per group were used to assess myelin damage. At least three mice per group were used for TEM analysis.

### 2.10. Statistical Analysis

All data in the present study are expressed as mean ± SEM. The statistical analyses were performed using SPSS 18.0 software (SPSS, Inc., Chicago, IL, United States). Behavioral data collected at repeating time points were analyzed using two-way ANOVA, followed by Turkey's post hoc test. For data with a single time point or drug dose, multiple comparisons were performed by one-way analysis of variance (ANOVA), and then multiple comparisons were performed using Turkey's post hoc test. A *p* < 0.05 was considered to be statistically significant.

## 3. Results

### 3.1. Ambroxol Promotes Functional Recovery and Attenuates ICH-Induced Brain Edema in Mice

**Figure 2:**
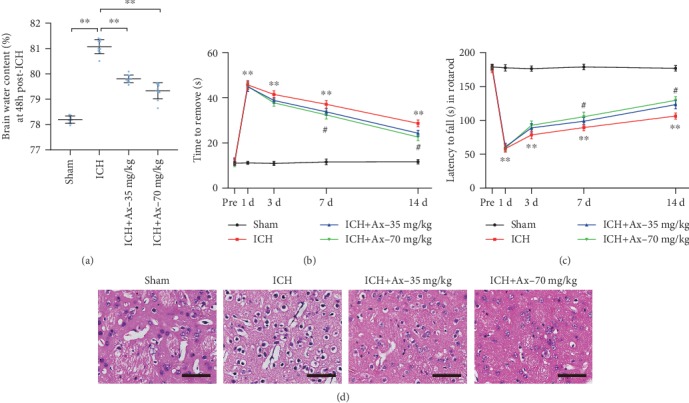
Ambroxol attenuates ICH-induced brain edema and promotes functional recovery in mice. (a) Brain water content, ^∗∗^*p* < 0.01, *n* = 6. (b) Adhesive removal test, ^∗∗^*p* < 0.01, *n* = 6. (c) Accelerated rotarod test, ^∗∗^*p* < 0.01, *n* = 6. (d) HE staining. Scale bar: 200 *μ*m and 20 *μ*m for enlarge images, respectively.

To uncover the effect of ambroxol on mice with ICH, two concentrations of ambroxol (35 mg/kg and 70 mg/kg) were performed to assess their benefit on functional recovery after ICH. The results showed that 35 mg/kg and 70 mg/kg ambroxol facilitated functional recovery using the rotarod test and adhesive removal test from day 3 to 14, compared with that in the ICH group (Figures 2(a) and 2(b)). Then, to reveal the reason why ambroxol enhances functional recovery, the brain water content on day 3 was assessed, and the results demonstrated that group of 35 mg/kg and 70 mg/kg ambroxol significantly reduced brain water content, compared with that in the ICH group (Figure 2(c)). Meanwhile, the HE staining on day 3 indicated that 35 mg/kg and 70 mg/kg ambroxol obviously decreased cell edema on day 3 post-ICH. Collectively, our results revealed that 35 mg/kg and 70 mg/kg ambroxol benefited functional recovery and reduced the brain water content and cell edema after ICH in mice.

### 3.2. Ambroxol Promotes Neuronal Survival in Mice with ICH

**Figure 3:**
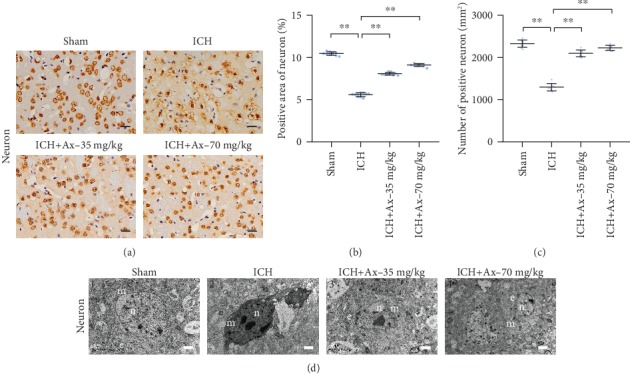
Ambroxol facilitates neuronal survival in mice with ICH. (a) The representative immunohistochemistry staining showed NeuN expression around the hematoma after ICH in different groups. Scale bar: 50 *μ*m. (b) Statistical graph for the number of NeuN^+^ cells per high power field according to the immunohistochemistry staining, ^∗∗^*p* < 0.01, *n* = 6. (c) Quantification of NeuN^+^ area per high power field according to the immunohistochemistry staining, ^∗∗^*p* < 0.01, *n* = 6. (d) Transmission electron micrographs (TEM) of neuron in ipsilateral basal ganglia from each group mouse brain tissue. Scale bars: ((d) 1 and 3) 1 *μ*m; ((d) 2 and 4) 2 *μ*m. n: nucleus; c: cytoplasm; e: endoplasmic reticulum, *n* = 6.

To further understand the reason why ambroxol benefited functional recovery and the outcome of reducing the brain water content and cell edema after ICH in mice, immunohistochemistry was employed to evaluate the number of neurons among lesions. The results showed that 35 mg/kg and 70 mg/kg ambroxol obviously increased the area and number of NeuN^+^, compared with that in the ICH group (Figures 3(a)–3(c)). Meanwhile, the ultrastructural characteristics using transmission electron microscopy demonstrated that 35 mg/kg and 70 mg/kg ambroxol enhanced the rehabilitation of neurons among lesions through protecting nuclear membrane against nuclear membrane fracture, inhibiting mitochondrial enlargement and chromatin atrophy (Figure 3(d)). Together, these results suggested that ambroxol helps promote neuronal survival among lesions in mice after ICH.

### 3.3. Ambroxol Reduces White Matter Fiber Bundle Damage in Mice with ICH

**Figure 4:**
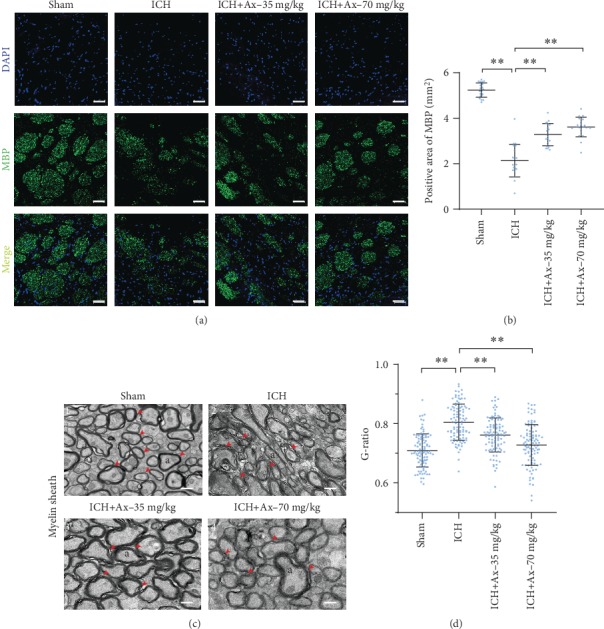
Ambroxol reduces white matter fiber bundle damage in mice with ICH. (a) The representative immunostaining showed MBP expression around the hematoma after ICH in different groups. Scale bar: 50 *μ*m. (b) Quantification of MBP^+^ area per high power field according to the immunostaining, ^∗∗^*p* < 0.01, *n* = 4. (c) Transmission electron micrographs (TEM) of myelin sheath in ipsilateral basal ganglia from each group mouse brain tissue. The red arrows mark the phenomenon of demyelination. TEM shows the myelin sheath around the hematoma are thinned and disintegrated post-ICH in mice. Scale bars: ((c) 1, 2, and 3) 500 nm; ((c) 4) 1 *μ*m. a: axon. (d) Statistical graph for G-ratio to evaluate the myelin sheath. ^∗∗^*p* < 0.01, *n* = 4.

Maintaining white matter fiber bundle integrity plays an essential role in functional exerting, except for intact neuronal network. These results indicated that ambroxol promoted neuronal survival. Hence, the effect of ambroxol on white matter fiber bundle was investigated. Our results revealed that 35 mg/kg and 70 mg/kg ambroxol reduced white matter fiber bundle damage in mice post-ICH on day 3 (Figures 4(a) and 4(b)). Furthermore, the ultrastructural characteristics using transmission electron microscopy depicted that 35 mg/kg and 70 mg/kg ambroxol facilitated axon uniformity (Figures 4(c) and 4(d)). Generally, our results indicated that ambroxol kept white matter fiber bundle integrity among lesions to promote functional recovery in mice after ICH.

### 3.4. Ambroxol Mitigates Microglial Activation and Reduces Proinflammatory Cytokine Accumulation

**Figure 5:**
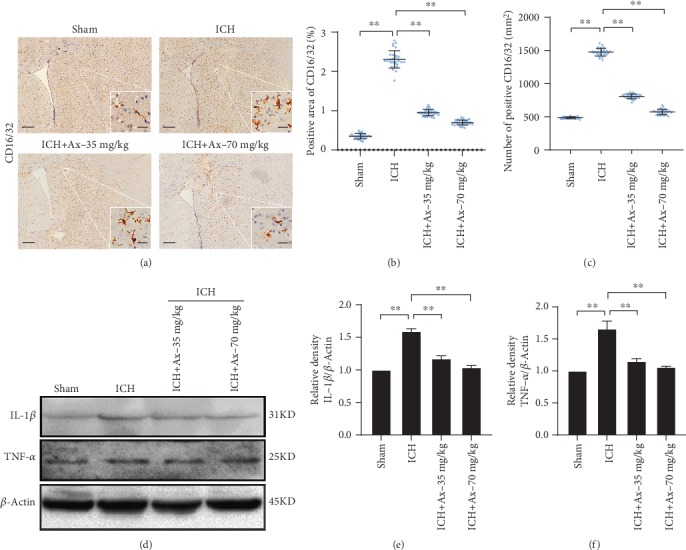
Ambroxol mitigates microglial activation and reduces proinflammatory cytokine accumulation. (a) The representative immunohistochemistry staining showed CD16/32 expression around the hematoma after ICH in different groups. Scale bar: 200 *μ*m and 20 *μ*m for enlarge images, respectively. (b) Statistical graph for CD16/32^+^ area cells per high power field according to the immunohistochemistry staining, ^∗∗^*p* < 0.01, *n* = 6. (c) Quantification of the number of CD16/32^+^ per high power field according to the immunohistochemistry staining, ^∗∗^*p* < 0.01, *n* = 6. (d) Representative bands showed the expression of IL-*β* and TNF-*α*. (e) Bar graph summarized the expression of IL-*β* from (d). ^∗^*p* < 0.05; NS: not significant, *n* = 4. (f) Quantification of the expression of TNF-*α* from (d). ^∗∗^*p* < 0.01, *n* = 4.

It is well known that neuroinflammation is involved in the pathogenesis of ICH and microglia are specialized macrophages residing in the central nervous system (CNS) [19]. Increasing evidence shows that it plays an essential role in neuroinflammation and activated microglia, especially M1-like type microglia, promotes proinflammatory factor accumulation, such as nitric oxide, tumour necrosis factor-*α* (TNF-*α*), and interleukin-1*β* (IL-1*β*), which exaggerate cell death and brain tissue injury [19, 20]. Thereafter, we employed immunohistochemistry to assess the M1-like type microglial activation, and the results depicted that 35 mg/kg and 70 mg/kg ambroxol reduced CD16/32^+^ microglial activation and decreased the number of CD16/32^+^ cells (Figures 5(a)–5(c)). At the same time, western blotting assays demonstrated that 35 mg/kg and 70 mg/kg ambroxol were able to decrease the TNF-*α* and IL-1*β* accumulation (Figures 5(d)–5(f)). Herein, our results indicated that ambroxol mitigated M1-like microglial activation and reduced proinflammatory cytokine accumulation.

### 3.5. Ambroxol Suppresses Endoplasmic Reticulum (ER) Stress in Microglia Involving the IRE1*α*/TRAF2 Signaling Pathway

**Figure 6:**
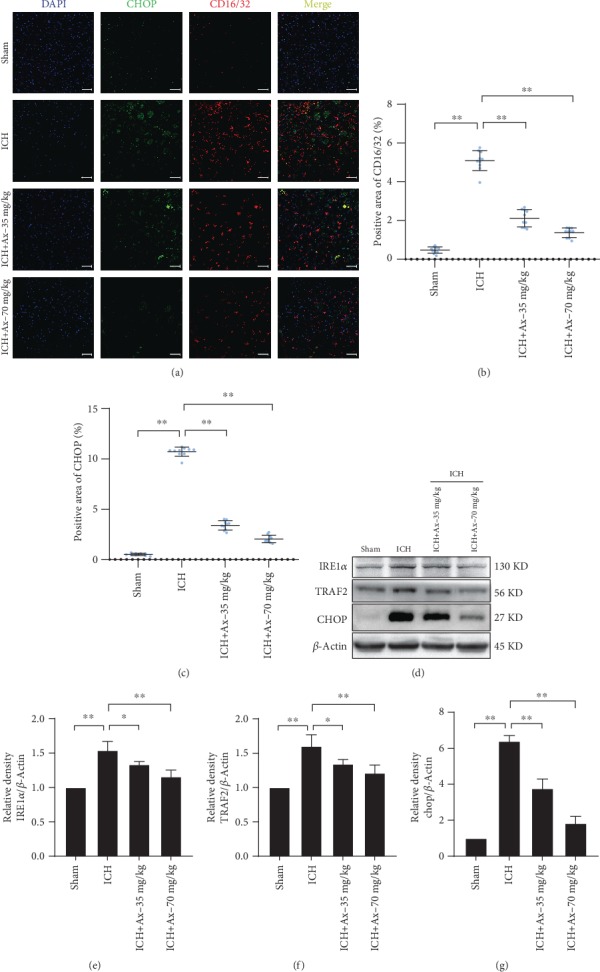
Ambroxol suppresses endoplasmic reticulum stress involving the IRE1*α*/TRAF2 signaling pathway. (a) The representative immunostaining showed colabelling of CHOP (green) and CD16/32 (red) around the hematoma after ICH in different groups. Scale bar: 50 *μ*m. (b) Statistical graph for CD16/32^+^ area cells per high power field according to the immunostaining, ^∗∗^*p* < 0.01, *n* = 6. (c) Quantification of the area of CHOP^+^ per high power field according to the immunostaining, ^∗∗^*p* < 0.01, *n* = 6. (d) Bands depicted the expression of IRE1*α*, TRAF2, and CHOP around the hematoma after ICH in different groups. (e) Bar graph summarized the expression of IRE1*α* from (d). ^∗^*p* < 0.05; ^∗∗^*p* < 0.01, *n* = 4. (f) Quantification of the TRAF2 expression of from (d). ^∗^*p* < 0.05; ^∗∗^*p* < 0.01, *n* = 4. (g) Quantification of the expression of CHOP from (d). ^∗∗^*p* < 0.01, *n* = 4.

ICH always disturbs the normal ER functions causing endoplasmic reticulum (ER) stress, which may subsequently lead to unfolded or misfolded ER proteins [21]. During this process, ER stress often manipulates microglial activation. Herein, to unravel why ambroxol suppressed M1-like microglial activation and decreased proinflammatory cytokine accumulation, immunofluorescence was used to determine CHOP expression in microglial after ICH. The results revealed that 35 mg/kg and 70 mg/kg ambroxol reduced the number of CD16/32^+^ cells and the expression of CHOP among lesions, compared to the ICH group (Figures 6(a)–6(c)). Then, western blotting assays were carried out to certify the results obtained from immunofluorescence. The bands depicted that 35 mg/kg and 70 mg/kg ambroxol downregulated IRE1*α*, TRAF2, and CHOP expression (Figures 6(d)–6(g)). In general, ambroxol represses endoplasmic reticulum (ER) stress in microglia involving the IRE1*α*/TRAF2 signaling pathway.

## 4. Discussion

In the present study, our results certified our hypothesis that ambroxol benefits functional recovery in mice with ICH. The data showed that ambroxol mitigated M1-like microglial activation and reduced proinflammatory cytokine accumulation (TNF-*α* and IL-1*β*) via suppressing ER stress to facilitate neuronal survival and to reduce white matter fiber bundle damage and finally promoting functional recovery.

Microglia are the main resident immune cells in the brain. When activated, microglia/macrophages are associated with the proinflammatory M1 phenotype, resulting in the release of multiple proinflammatory cytokines, such as nitric oxide, TNF-*α*, and IL-1*β*, which exaggerate cell death and brain tissue injury [19, 20]. Our results found that ambroxol mitigated M1-like microglial activation and reduced TNF-*α* and IL-1*β* accumulation. Previous study has revealed that TNF-*α* mediates endothelial necroptosis aggravating blood-brain barrier disruption after ischemic stroke [20], which might explain the reason why ambroxol relieves brain water content in the present work. Furthermore, TNF-*α* reduction might also contribute to the increasing number of neurons and axons among lesions to enhance functional recovery due to hindering neural cell loss. In addition, some of the activated microglia might polarize into M2-like phenotype with ambroxol treatment. Previous studies have indicated that the transition from the M1 to the M2 phenotype leads to brain repair and regeneration after brain injury, such as ischemia, ICH, neurodegenerative diseases, and traumatic brain injury (TBI) [22–24].

Endoplasmic reticulum (ER), which is responsible for the proper folding and assembly of most secretory and membrane proteins, is an essential organelle in eukaryotic cells [25]. ICH always disturbs the normal ER functions and subsequently causes ER stress resulting in unfolded or misfolded ER proteins [21]. Unfolded protein response (UPR) facilitates ER function integrity and helps restore protein folding homeostasis at the initial phase, while it also triggers apoptosis if ER stress remains unmitigated [26–28]. IRE1*α*, which is universally expressed in most tissues and cells, is one of the main signaling components to exaggerate cell apoptosis when activated [28]. Besides, IRE1*α* bears another function to cleave several mircoRNAs resulting in an enhancement in cell death [29, 30]. These evidences suggest that IRE1*α* downregulation helps maintain cell survival, which is consistent with our results that ambroxol promotes neuronal survival and reduces white matter fiber bundle damage in mice with ICH through downregulating IRE1*α*. Our data also indicated that TRAF2, one of the axis downstream molecules, was decreased with ambroxol administration, implying that IRE1*α*-TRAF2 axis was involved in ambroxol suppressing ER stress after ICH. Previous studies have demonstrated that the cyclophosphamide, doxorubicin, vincristine, and prednisone (CHOP) can induce transcriptional activation of genes that ultimately lead to cell death [31, 32]. Moreover, recent study has proven that CHOP silencing attenuates acute brain injury in rats after subarachnoid hemorrhage (SAH) [33], which is in line with our data that the expression of CHOP among lesions was downregulated when using ambroxol, compared to the ICH group. In addition, studies have indicated that IRE1*α*-TRAF2 axis manipulates ASK1 complex to elicit activation of p38 mitogen-activated protein kinase signaling pathways [34, 35], which results in CHOP activation and leads to cell loss. Although PKR-like ER protein kinase (PERK) and activating transcription factor 6 (ATF6) are other two UPR classes of signaling components, we focus on IRE1*α*-TRAF2 axis signaling cascade in the present research. Whether other signaling factors are involved in this process needs to be certified in our future work.

About 43% of all ICH patients suffering from pneumonia, which is significantly influencing outcome and accounting for more than 1/3 of the overall mortality in patients with ICH [3, 4]. Previous studies have indicated that ambroxol significantly decreased the bronchopulmonary complications after upper abdominal chest and cardiac surgeries [10, 36]. Most recently, a study has demonstrated that ambroxol could reduce lung infection resulting from severe cervical spinal cord injury (CSCI) through decreasing airway secretion and release of inflammatory material [37]. Hence, ambroxol may be an effective additional treatment for ICH patients. The potential mechanism might due to anti-inflammation and antioxidant effects in addition to the mucokinetic and mucociliary effects of the parent compound [10]. Furthermore, a study has shown that ambroxol could effectively cross the BBB and has no harmful effect even at high doses. Here, our results certified that 35 mg/kg and 70 mg/kg ambroxol facilitated neuronal survival and reduced white matter fiber bundle damage due to mitigating M1-like microglial activation and reducing proinflammatory cytokine accumulation in mice with ICH. To our limited knowledge, this research firstly reveals the role of ambroxol in ICH treatment, even in other brain traumatic injury (TBI), which opens a new avenue for the use of ambroxol in ICH treatment.

There are still some issues need to be answered in our next investigation. First, whether ambroxol facilitates the transition of microglia from the M1 to M2 phenotype needs to be elucidated in our future work. Next, whether other signaling pathway except for IRE1*α*/TRAF2 is involved in ambroxol promoting functional recovery post-ICH in mice needs to be determined. Meanwhile, ambroxol, serving as a GCase chaperone, helps increase GCase activity, which is the enzyme responsible for breaking down glucocerebroside into glucose and ceramide in the lysosome [38], to regulate the lysosomal pathway and autophagy pathway benefiting neural survival in Parkinson's disease [39]. At the same time, neurotrophic niche is another factor affecting neural survival. Hence, the role of the lysosomal pathway, autophagy pathway, and trophic signaling pathway playing in ambroxol promoting neuronal survival and which one plays the leading role need to be clarified in our future work. In addition, to improve the effectiveness of ambroxol administration, the duration and method should be explored in our next research.

## 5. Conclusions

In short, our present study has uncovered a pivotal role of ambroxol in ICH except for pneumonia treatment. Meanwhile, the IRE1*α*/TRAF2 signaling pathway is involved in ambroxol facilitating neuronal survival and reducing white matter fiber bundle damage due to mitigating microglial activation and decreasing proinflammatory cytokine accumulation in mice with ICH, which paves a new path for the treatment of ICH and opens a new window for the use of ambroxol in clinical practice.

## Data Availability

The data used to support the findings of this study are available from the corresponding author upon reasonable request.
