# Corrigendum to “Ambroxol Improves Neuronal Survival and Reduces White Matter Damage through Suppressing Endoplasmic Reticulum Stress in Microglia after Intracerebral Hemorrhage”

**DOI:** 10.1155/2020/7941716

**Published:** 2020-11-30

**Authors:** Xuheng Jiang, Ji Zhang, Bojin Kou, Chao Zhang, Jun Zhong, Xuanyu Fang, Xiaofei Huang, Xiaojun Zhang, Fangke Xie, Quan Hu, Hongfei Ge, Anyong Yu

**Affiliations:** ^1^Department of Emergency, Affiliated Hospital of Zunyi Medical University, 563003 Zunyi, Guizhou, China; ^2^Department of Neurosurgery and Key Laboratory of Neurotrauma, Southwest Hospital, Third Military Medical University (Army Medical University), 400038 Chongqing, China

In the article titled “Ambroxol Improves Neuronal Survival and Reduces White Matter Damage through Suppressing Endoplasmic Reticulum Stress in Microglia after Intracerebral Hemorrhage” [1], there was an error in the University name in affiliation 1. The correct affiliation should be “Affiliated Hospital of Zunyi Medical University,” instead of “The First Affiliated Hospital of Zunyi Medical University.” These details are corrected in the author information above.
